# Altered dental plaque microbiota correlated with salivary inflammation in female methamphetamine users

**DOI:** 10.3389/fimmu.2022.999879

**Published:** 2022-11-28

**Authors:** Zhuohang Deng, Kaili Guo, Fengdi Cao, Tiantian Fan, Bin Liu, Mingyue Shi, Yue Liu, Zhe Ma

**Affiliations:** Department of Preventive Dentistry, Hebei Key Laboratory of Stomatology, Hebei Clinical Research Center for Oral Diseases, School and Hospital of Stomatology, Hebei Medical University, Shijiazhuang, China

**Keywords:** methamphetamine abuse, supragingival dental microbiota, 16S rRNA sequencing, oral health, salivary inflammation, 14-item oral health impact profile (OHIP-14)

## Abstract

Poor oral health is the most immediate and overlooked hazard of methamphetamine abuse in humans. Previous studies have reported methamphetamine-associated alterations in saliva microbiota, but the cause of methamphetamine-induced alterations in the oral microenvironment remains unclear. The present study aimed to investigate the alterations in dental plaque microbiota in methamphetamine users, and to explore their relationship with local immune inflammation in the oral cavity. This may provide new ideas on the development of methamphetamine-related oral microenvironment changes. Questionnaires and samples were obtained from 30 female methamphetamine users and 15 sex- and age-matched healthy controls. Microbial profiles of supragingival dental plaque were analyzed using 16S rRNA gene sequencing. Inflammatory factors in saliva were measured using enzyme-linked immunosorbent assay. Methamphetamine users had worse oral self-evaluation. Compared with healthy controls, methamphetamine users showed no differences in oral dental plaque microbial diversity but exhibited differences in the relative abundance of several microbial taxa. At the phylum level, a higher abundance of Proteobacteria and a lower abundance of Firmicutes were detected in methamphetamine users. Moreover, function prediction using the MetaCyc database showed that 33 pathways were significantly upregulated in methamphetamine users; Only the glycolytic (Pyrococcus) pathway was enriched in the C group. Importantly, salivary inflammatory factors showed complex significant associations with bacterial genera in methamphetamine users. Specifically, the genus *Neisseria* was positively correlated with IL-17 levels in saliva, and both were high in methamphetamine users. In contrast, the genus *Strept*ococcus, with a lower abundance, was positively correlated with lower IL-10 levels. Overall, This study is the first to provide evidence for a link between altered dental plaque microbiota and salivary inflammation in methamphetamine users. Further elucidation of the interactions between methamphetamine use and oral microenvironment would be beneficial for appropriate interventions to improve oral health.

## 1 Introduction

Drug abuse is a major public hazard worldwide and has become a global social and public health problem that has attracted increasing attention. According to the World Drug Report 2021, released by the United Nations Office on Drugs and Crime (UNODC), approximately 275 million people worldwide used drugs in 2020 (1). Drug consumption has continued to increase globally during the COVID-19 pandemic. The United Nations Drug Control Agency statistics show that the number of methamphetamine users ranks second worldwide, and with China ranking first in this number. Therefore, the harmful effects of methamphetamine cannot be underestimated.

Previous studies have demonstrated that methamphetamine causes dysfunction of the central nervous, immune, and cardiovascular systems as well as affects the occurrence and development of oral diseases. Oral health is one of the most frequently reported health problems among methamphetamine users, and is also the most direct and easily ignored effect of methamphetamine on the human body. “Meth mouth” specifically occurs in methamphetamine users and is characterized by extensive and serious dental caries damage (2). Further, the incidence of periodontal disease, xerostomia, and oral mucosal diseases is also higher in methamphetamine users than that in healthy people (3). The etiology of methamphetamine-associated oral disease is unknown, but the current view is based on the interaction of multiple factors. These oral diseases may be related to the change in living and eating habits, poor oral hygiene, endocrine dysfunction, smoking and other factors (4). However, the mechanism of action and systemic physiological effects of methamphetamine are also essential. For instance, methamphetamine is acidic, and smoking it has a direct corrosive effect on oral tissues (5). Dry mouth caused by methamphetamine is extremely common and can promote the development of serious caries and periodontal disease (6).

The concept of systemic immune function inhibition by methamphetamine is a few decades old. Methamphetamine can change cellular components and restrain the ability of immune cells (7), increase the release of inflammatory molecules (cytokines, chemokines, and cell adhesion molecules), and decrease anti-inflammatory cytokines (8). Deng D et al. (9)provided evidence for a link between altered fecal microbiota and systemic inflammation in methamphetamine use disorder. Yang Y and Yu X et al. (10) found that methamphetamine users had a significantly lower species richness, and exhibited higher relative abundance in several bacterial taxa that were known to be related to oral diseases in saliva microbiota. In our study, we focused on the changes in the local microenvironment of the oral cavity, including the microbiota and the immuno inflammation of saliva. Thus, upon detection of the alterations in the microbiota composition of methamphetamine users, we hypothesized that such differences may arise from changes alterations in the immune microenvironment caused by methamphetamine.

Further, the oral cavity is a unique ecosystem that includes the teeth, gingival groove, tongue, cheek, and soft and hard palate. Each adult mouth contains, on average, approximately 5–100 billion bacteria, representing approximately 200 major bacterial species (11). Alterations in the oral microflora have been observed in dental caries, periapical infection, periodontal disease, halitosis, oral ulcers, and other oral diseases. Oral microbiota composition is increasingly believed to be related to diseases in other parts of the body or to reflect systemic health. In this case, the oral microbiota profile can be used as a diagnostic biomarker for other diseases such as Alzheimer’s syndrome (12), diabetes (13), coronary heart disease (14), and rheumatoid arthritis (15). In the diseased state, the oral microbial community undergoes ecological transformation in response to host homeostasis disorders (16). Recently, methamphetamine-associated alterations in saliva microbiota had been reported. However, the determinants of these microbial imbalances are largely unknown, and the association between the oral microbiota and local environment in methamphetamine users remains unclear.

Meanwhile, microbes differ in different parts of the mouth, including the saliva, tongue, oral mucosa, mineralized tooth surfaces, and periodontal tissue, each of which has a unique microbial community (17), with dental plaque being the most abundant and diverse (18). Dental biofilms are functional and structurally ordered communities comprising synergistic or antagonistic microorganisms, which can prevent the colonization of non-oral microorganisms and provide protection to the surface of teeth. Therefore, the present study aimed to investigate alterations in the supragingival dental plaque microbiota and explore its relationship with salivary inflammation in methamphetamine users.

## 2 Materials and methods

### 2.1 Subject selection

The study included 30 women who used methamphetamine in Shijiazhuang, Hebei Province, China, between March 2021 and December 2021and 15 sex- and age-matched healthy controls. The 30 methamphetamine users were defined as group M and 15 healthy people as group C. All participants signed an informed consent form, all personal information was kept confidential, and the study was approved by the Medical Ethics Committee of the School and Hospital of Stomatology, Hebei Medical University (Shijiazhuang, Hebei, China). The participants in group M were clinically diagnosed with methamphetamine use disorder (MUD) and there was limited confounding by the use of other illicit drugs, such as heroin, cocaine, and cannabis. group C, with no history of neuropsychiatric conditions or illicit substance use, was recruited randomly. For the two groups, the inclusion criteria for all participants were as follows: not pregnant or lactating, aged between 20 and 50 years, and total number of teeth ≥ 20. Additional exclusion criteria were as follows: (1) chronic medical conditions, infectious diseases, genetic diseases, and autoimmune diseases; (2) use of antibiotics, probiotics, corticosteroids, or any other immunomodulators within three months before sample collection; (3) experience of open surgery on the head or mouth; and (4) symptoms of acute inflammation, infection, or trauma.

### 2.2 Questionnaire data collection and analysis

To understand the basic information and oral health status of drug users, in accordance with the World Health Organization (WHO), Basic Methods of the Oral Health Survey, and 4th National Oral Health Epidemiological Survey Standards, combined with the actual situation of the methamphetamine use disorder (MUD), we set up an oral disease investigation project. Additionally, the occurrence and development of methamphetamine-related oral diseases affects the face, chewing, and pronunciation of drug users. These damages also restrict the patients’ daily life and social activities to varying degrees, thus indirectly affecting the psychological state and social activities of patients. The 14-item Oral Health Impact Degree Scale (OHIP14) was used to compare the oral health-related quality of life (OHRQoL) between the M and C groups. The version of OHIP14 was translated by Weini Xin into Chinese, with a Cronbach’s coefficient of 0.93 (19). This scale contains 14 items in four dimensions, using the Likert level 5 scoring method, with each item scoring 0–4 points, out of 56 points. The higher the score, the worse the oral health-related quality of life, with a cut-off value of 12 points. All participants were asked to complete an on-site questionnaire and the 14-item Oral Health Impact Degree Scale (OHIP14). Comparisons of these information between the two groups were analyzed using SPSS software (v26.0, IBM Statistics, Chicago, IL, USA). Continuous variables were tested using Mann-Whitney U test depending on the distribution normality evaluated by Shapiro-Wilk’s test. Categorical variables were examined by Pearson Chi-square test or Continuity Correction test. P value (two-sided) < 0.05 was considered significant.

### 2.3 Inflammatory indicators, sample collection, and methods

The two groups of participants gargled with water in the morning, dispensed 1.5 ml of non-stimulated saliva in microcentrifuge tubes. The saliva samples were then centrifuged at 3000 rpm for 10 min, and the supernatant was stored at -80°C refrigerator. The levels of cytokines IL-1, IL-6, TNF-α, IL-17, and IL-10 in the saliva of the two groups were measured using enzyme-linked immunosorbent assays purchased from the Jiangsu Jingmei Company (China). Data on inflammatory factor levels conformed to normal distribution by Shapiro-Wilk’s test, and were tested using Student’s t test. P value (two-sided) < 0.05 was considered significant.

### 2.4 16s rRNA gene sequencing methods

#### 2.4.1 Sample collection and DNA extraction

Plaque samples were collected from both groups in the early morning before any oral hygiene practice. The subjects were asked to refrain from eating or drinking for at least 3 h prior to sample collection. The sampling operator rinsed the dental surfaces of the subjects with sterile saline, dried the collected teeth with cotton rolls, then collected the buccal, lingual and fossa plaque of the anterior and posterior teeth in the four quadrants of the oral cavity with a sterile oral probe, and collected the interproximal plaque with dental floss. Plaque was collected in sterile DNAand RNA-free Eppendorf tubes and stored at −80°C until use. Total genomic DNA samples were extracted from the supragingival dental plaque using the OMEGA Soil DNA Kit (M5635-02) (Omega Bio-Tek, Norcross, GA, USA), following the manufacturer’s instructions, and stored at -20 ℃ prior to further analysis. The quantity and quality of extracted DNAs were measured using a NanoDrop NC2000 spectrophotometer (Thermo Fisher Scientific, Waltham, MA, USA) and agarose gel electrophoresis, respectively.

#### 2.4.2 16S rRNA gene amplicon sequencing

Specific primers with barcodes (16S V3-V4:338F and 806R) were used for PCR amplification of the bacterial 16S rRNA gene. Sample-specific 7-bp barcodes were incorporated into the primers for multiplex sequencing. The PCR components contained 5 µl of buffer (5×), 0.25 µl of Fast Pfu DNA Polymerase (5U/µl), 2 µl (2.5 mM) of dNTPs, 1 µl (10 µM) of each forward and reverse primer, 1 µl of DNA template, and 14.75 µl of ddH_2_O. Thermal cycling consisted of initial denaturation at 98°C for 5 min, followed by 25 cycles of denaturation at 98°C for 30 s, annealing at 53°C for 30 s, and extension at 72°C for 45 s, with a final extension of 5 min at 72°C. PCR amplicons were purified using Vazyme VAHTSTM DNA Clean Beads (Vazyme, Nanjing, China) and quantified using the Quant-iT PicoGreen dsDNA Assay Kit (Invitrogen, Carlsbad, CA, USA). After the individual quantification step, amplicons were pooled in equal amounts, and paired-end 2× 250 bp sequencing was performed using the Illumina NovaSeq platform with the NovaSeq 6000 SP Reagent Kit (500 cycles) at Shanghai Personal Biotechnology Co., Ltd (Shanghai, China).

#### 2.4.3 Sequence analysis

Microbiome bioinformatics was performed using QIIME2. Briefly, raw sequence data were demultiplexed using the demux plugin, followed by primer cutting using the cutadapt plugin. Sequences were then quality-filtered, denoised, merged, and chimera removed using the DADA2 plugin. Non-singleton amplicon sequence variants (ASvs) were aligned with mafft and used to construct a phylogeny using fasttree2. Alpha and beta diversity metrics were estimated using the diversity plugin with the samples. Taxonomy was assigned to the ASvs using the classify-sklearn naïve Bayes taxonomy classifier in the feature-classifier plugin against the HOMD_16S database (Human Oral Microbiome Database).

#### 2.4.4 Bioinformatics and statistical analysis

Sequence data analyses were mainly performed using the QIIME2 and R packages (v3.2.0). ASV-level alpha diversity indices, such as the Chao1 richness estimator, observed species, Shannon diversity index, Simpson index, Faith’s PD, Pielou’s evenness, and Good’s coverage were calculated using the ASV table in QIIME2 and visualized as box plots. ASV-level ranked abundance curves were generated to compare the richness and evenness of ASvs among samples. Beta diversity analysis was performed to investigate the structural variation in microbial communities across samples using UniFrac distance metrics and visualized *via* principal coordinate analysis (PCoA). The significance of differences in the microbiota structure among groups was assessed by permutational multivariate analysis of variance (PERMANOVA) using QIIME2. The difference test boxplot shows the p-value of the total difference between the groups of a species obtained using the Kruskal-Wallis non-parametric test and the significance level markers of the difference obtained by paired comparison in Dunn’s test between groups. A Venn diagram was generated to visualize shared and unique ASvs among samples or groups using the R package “VennDiagram,” based on the occurrence of ASvs across groups, regardless of their relative abundance. Linear discriminant analysis effect size (LEfSe) was used to detect differentially abundant taxa across groups using default parameters. Microbial functions were predicted using Phylogenetic investigation of communities by reconstruction of unobserved states (PICRUSt2) in the MetaCyc (https://metacyc.org/) database. The Spearman test method was used to analyze the correlation between genus level species and salivary inflammation indices in all samples, and redundancy analysis (RDA) were used for mapping.

## 3 Results

### 3.1 Questionnaire survey and oral health impact profile

30 female methamphetamine users and 15 sex- and age-matched healthy controls were enrolled in this study. Demographic and clinical characteristics were listed in **Supplementary Table 1**. There is no significant difference between group M and group C in age, brushing habits and most oral self-assessment items (such as loose teeth, (food debris) stuck between teeth, bleeding, swelling, swollen gums and toothache)(all p > 0.05). However, the educational level of group M was significantly lower than that of group C (p = 0.01). And a greater proportion of methamphetamine users had a smoking habit (p < 0.001). Moreover, methamphetamine users had significantly more oral ulcers, bad breath and dry mouth than the healthy control individuals (p < 0.01). Contrary to expectation, OHIP-14 scores between the two groups were no significant difference (**Table 1** **)**, which were related to subjective perception.

**Table 1:** OHIP-14 (14-item Oral Health Impact Degree Scale)scores of the study participants.

Dimension	Items	Group M	Group C	P value
Pain and discomfort	3	3.00 (5.00)	3.00 (4.00)	0.223
Psychological discomfort	3	2.50 (6.50)	3.00 (4.00)	0.706
Functional restriction	3	3.00 (2.50)	3.00 (2.50)	0.289
Independence weakened	5	1.50 (6.25)	2.00 (2.00)	0.715
Total	14	10.00 (11.50)	13.00 (5.00)	0.647

Data are presented as Median (IQR, inter-quartile range), P values based on Mann-Whitney U test.

### 3.2 Dental plaque bacterial community structure differs between groups

**Figure 1:**
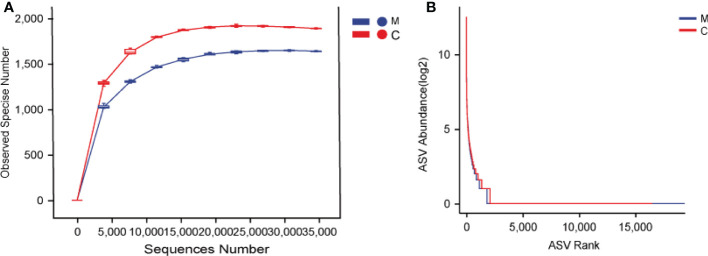
Dental plaque bacterial community structure differs between groups. **(A)** Rarefaction curves, **(B)** Rank abundance curves. The M and C groups are colored in blue and red, respectively.

We analyzed the supragingival dental plaque microbiota using 16S rRNA gene sequencing. The rarefaction curves of the two groups reached a plateau with an increase in the ordinate sequencing volume, which was sufficient to reflect the diversity of the current samples; the sequencing depth was sufficient to reflect the actual situation of oral microbes in the participants. In addition, slightly fewer species were observed in group M than in group C (**Figure 1A**). The rank abundance curves showed that the two groups of samples showed similar and steep broken lines, and that the abundance of each ASV in the community was differed significantly (**Figure 1B**). There were few high-abundance species, whereas most species showed extremely low abundance, presenting a “long-tail distribution.” Chao1, observed species, Shannon, Simpson, Faith ‘s pd, Pielou’s evenness, and Good’s coverage were used for alpha diversity analysis; the results showed that the M and C groups showed no significant differences in diversity (**Table 2**).

**Table 2:** Comparison of species alpha diversity based on ASV levels.

Group	Chao1	Faith_pd	Goods_coverage	Observed_species	Pielou_e	Shannon	Simpson
M	1638.58 ± 330.19	126.98 ± 29.G12	0.99 ± 0.002	1411.91 ± 315.80	0.65 ± 0.06	6.80 ± 0.85	0.95 ± 0.04
C	1838.26 ± 524.92	121.03 ± 25.98	0.99 ± 0.003	1690.84 ± 538.49	0.67 ± 0.07	7.13 ± 1.03	0.95 ± 0.04

Wilcoxon rank-sum test. All indices were analyzed using ASvs for different samples.

### 3.3 Dental plaque microbiota composition is altered in group M

**Figure 2:**
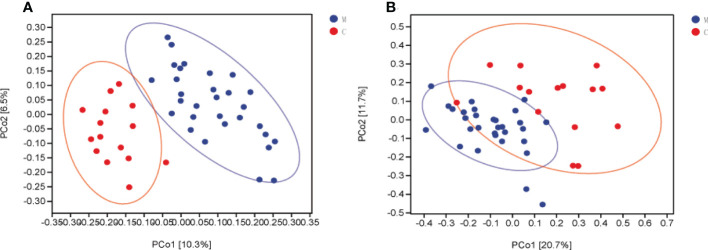
Dental plaque microbiota composition is altered in group M (methamphetamine using). **(A, B)** Principal coordinate analysis (PCoA) of bacterial beta diversity based on unweighted **(A)** and weighted **(B)** UniFrac distances.

Bacterial beta diversity was calculated to compare microbial community composition between the groups. Dental plaque microbiota in the two groups could be divided into clusters according to community composition in principal coordinate analysis (PCoA) based on weighted and unweighted UniFrac distances (**Figures 2A, B**). Permutation multivariate ANOVA results confirmed significant differences in the microbiota community composition between the two groups (**Supplementary Table 2**). To complement the clustering results, the Venn plot showed a total of 2,631 ASvs between the groups, 16749 ASvs unique to group M, and 13844 ASvs unique to group C (**Supplementary Figure 1**).

### 3.4 Different bacterial taxa are present between groups

**Figure 3:**
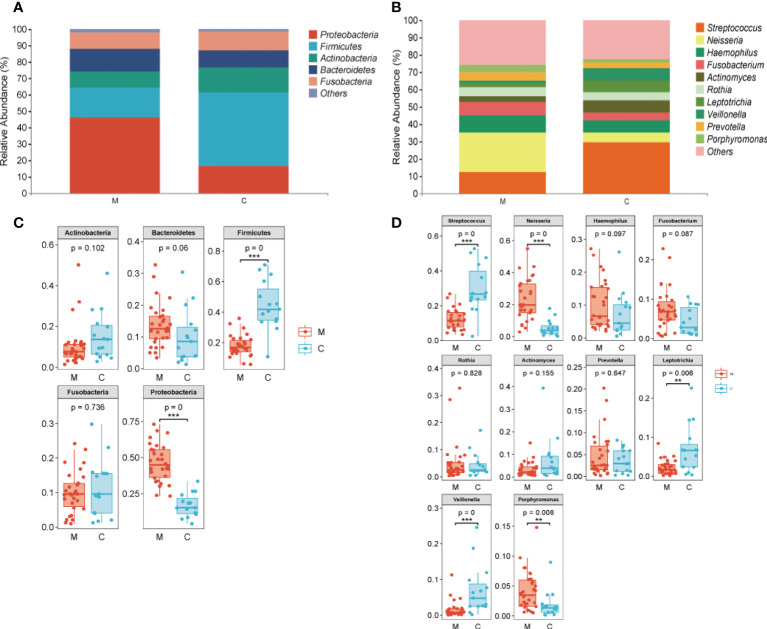
Taxa showing differences in relative abundance between the methamphetamine using (M) and control (C) groups. **(A)** Top five in abundance at the phylum level, **(B)** Top 10 in abundance at the genus level, **(C)** Difference test box diagram at the phylum level, **(D)** Difference test box diagram at the genus level. *p < 0.05, **p < 0.01, ***p < 0.001.

**Figure 4:**
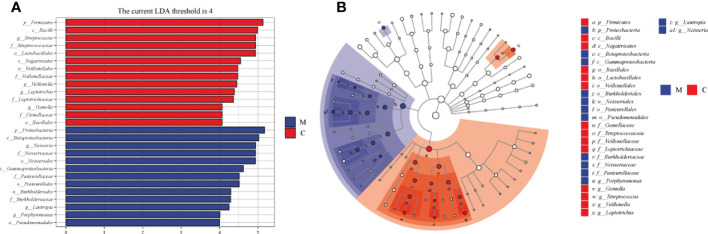
Differences in dental plaque microbiota composition between the methamphetamine using (M) and control (C) groups. **(A)** Cladograms generated using LEfSe showing taxonomic differences between groups M and C. Nodes in blue and red represent taxa that are less and more abundant in group M relative to those in group C. **(B)** Linear discriminant analysis (LDA) scores for bacterial taxa differing in abundance between groups M and C. Positive and negative LDA scores indicate taxa enriched in groups M and C, respectively. Only taxa with P < 0.01 (Wilcoxon rank-sum test) and LDA > 4.0 are shown.

The overall microbial compositions of the M and C groups were examined at different taxonomic levels, and we focused analyzing the oral microbial community distribution at the phylum and genus levels using a bar chart. At the phylum level, the microbial compositions of the two groups were similar, but their relative abundances differed (**Figure 3A**). Among the five dominant phyla, the relative abundances of Proteobacteria (46.19% *vs*. 16.93%, P < 0.001) and Bacteroidetes (13.68% *vs*. 10.10%) in group M were increased, whereas the relative abundances of Firmicutes (17.92% *vs*. 44.56%, P < 0.001), Actinobacteria (10.40% *vs*. 15.42%), and Fusobacteria (10.06% *vs*. 11.34%) were lower than those in group C (**Figure 3B**). Furthermore, the relative abundances of the top 10 bacterial genera varied between the groups (**Figure 3C**). Compared to those in group C, six genera had a higher relative abundance in group M: *Neisseria* (23.11% *vs*. 5.36%, P < 0.001), *Porphyromonas* (4.10% *vs*. 1.88%, P < 0.001), *Prevotella* (4.68% *vs*. 3.54%), *Rothi* (5.15% *vs*. 4.37%), *Fusobacterium* (7.52% *vs*. 4.38%), and *Haemophilus* (10.03% *vs*. 7.13%). The other four genera, *Veillonella* (1.58% *vs*. 6.96%, P < 0.001), *Streptococcus* (12.32% *vs*. 29.72%, P < 0.001), *Leptotrichia* (2.25% *vs*. 6.94%, P < 0.01), and *Actinomyces* (3.35% *vs*. 7.47%) decreased (**Figure 3D**). LEfSe analysis was performed to further analyze the bacterial community structure (**Figure 4A**), and linear discriminant analysis (**Figure 4B**) was used to estimate differences in the effect size of each taxon in the two groups. The results of LEfSe analysis illustrated that the relative abundances of Firmicutes, Bacilli, Negativicutes, Bacillales, Lactobacillales, Veillonellales, Gemllaceae, Leptotrichiaceae, Streptococcaceae, Veillonellaceae, Veillonella, Gemella, Streptococcus, and Leptotrichia were reduced, whereas those of Proteobacteria, Betaproteobacteri, Gammaproteobacteria, Burkholderiales, Pasteurellales, Neisseriales, Pseudomonadales, Neisseriaceae, Burkholderiaceae, Pasteurellaceae, Lautropia, Neisseria, and Porphyromonas were increased in group M compared to those in group C (**Figure 4**).

### 3.5 Functional properties of dental plaque microbiota predicted by PICRUSt2

**Figure 5:**
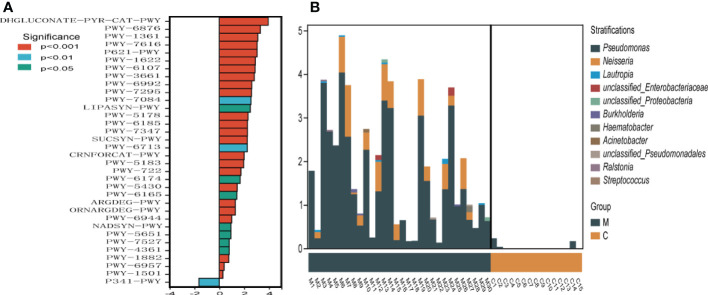
Metabolic pathway difference analysis **(A)**, positive value of logFC (log2(fold change)) on the horizontal axis represents upregulation in group M (methamphetamine using) compared with that in group C (control), whereas the negative value represents downregulation. The ordinates indicate different Pathway tags. **(B)** Species composition diagram of the glucose degradation (oxidative) metabolic pathway.

To understand the functional potential of the supragingival dental plaque microbiota, PICRUSt2 was used to compare existing 16SrRNA gene sequencing data using MetaCyc. A total of 34 pathways were identified with significantly different abundance in the dental plaque microbiota between the two groups. Compared with group C, group M was significantly upregulated in 33 pathways, including carbohydrate synthesis and degradation (glucose degradation, sucrose synthesis, arabinose degradation IV and 1, 5-anhydrous fructose degradation), amino acid synthesis and metabolism, and energy metabolism (creatinine degradation, nitrifier denitrification). Only the glycolytic (Pyrococcus) pathway was enriched in the C group (**Figure 5A**). Among the different metabolic pathways between the two groups, upregulation of glucose degradation was the most obvious in group M. The species composition diagram of the metabolic pathways showed that more species, mainly belonging to *Pseudomonas* and *Neisseria*, were involved in glucose degradation. Further, their abundance was relatively high in group M. However, very few species were involved in glucose degradation in group C (**Figure 5B**).

### 3.6 Relationships between altered dental plaque microbiota and salivary inflammation

**Figure 6:**
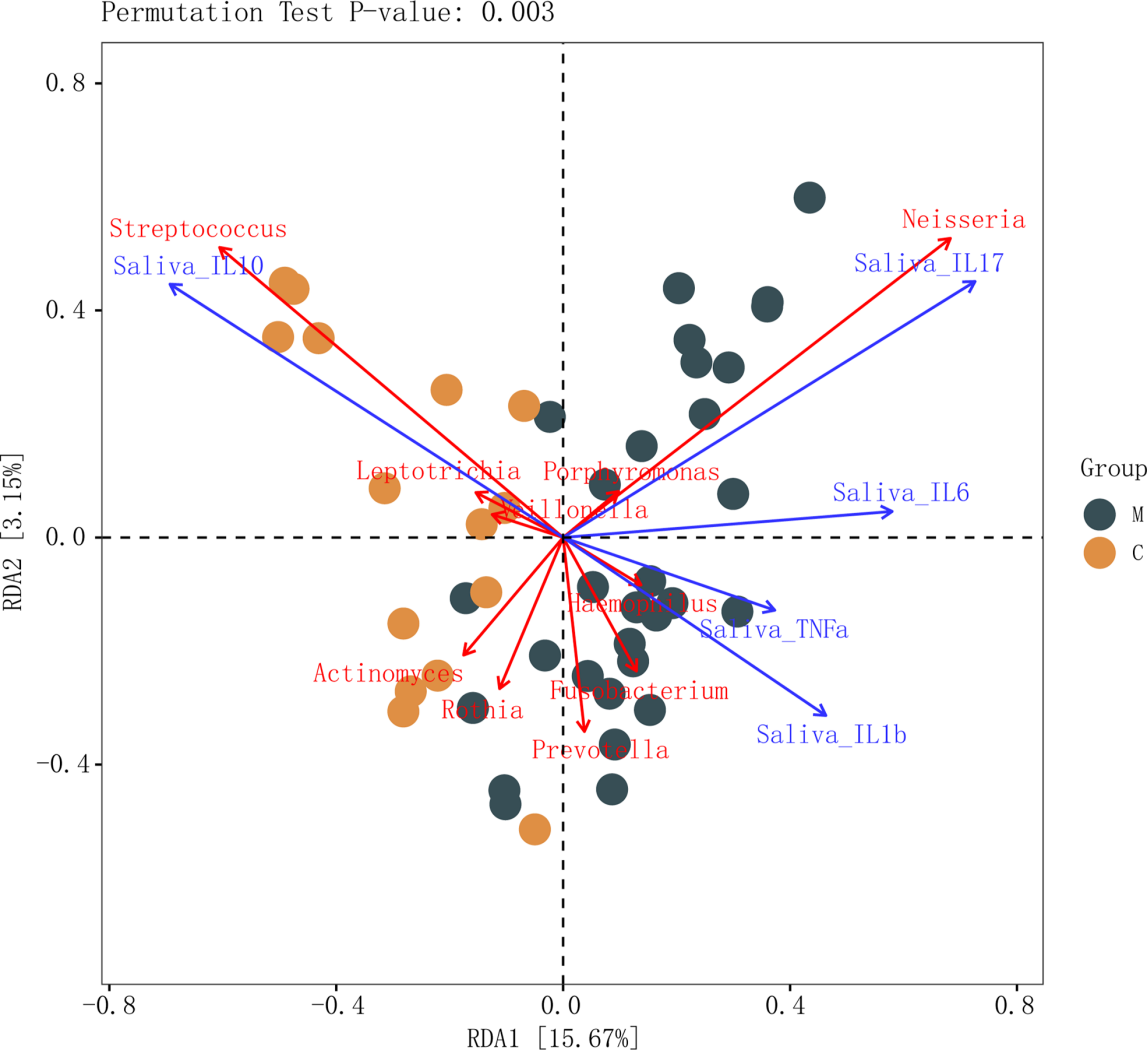
Connections between dental plaque microbiota and systemic immune inflammation. Redundancy analysis of inflammatory factors in the saliva and the top 10 genera in abundance in the methamphetamine using (M) and control (C) groups, permutation test P-value: 0.003. Each point represents a sample, different colored points belong to different groups. Blue arrows represent different inflammatory factors, red arrows represent bacterial genera, the angle between arrows represents the size of the correlation between them.

The results of salivary inflammatory factor levels indicated that the levels of IL-1β and IL-17 in the saliva of group M were significantly higher than those in group C, and the levels of IL-6 and TNF-α tended to increase. However, the IL-10 level was significantly lower than that in group C (**Table 3**). In the redundancy analysis (RDA), the lengths of IL-10 and IL-17 rays were the longest, indicating that they had a significant impact on the structure of the oral microflora. Moreover, *Neisseria* was positively correlated with IL-17, and the relative abundance of *Neisseria* was higher in group M. *Streptococcus* was positively correlated with IL-10, and its relative abundance was higher in group C (**Figure 6**). These results suggest a correlation between the structural alteration of the dental plaque microbiota and salivary inflammatory state of the host.

**Table 3:** Comparison of salivary inflammatory factors in the methamphetamine using (M) and control (C) groups.

	Group	Mean value	Std Dev	t value	df	P value
**IL-1β**	M	57.6	9.877	2.595	43	0.013^*^
	C	49.6	9.478			
**IL-6**	M	44.217	8.032	1.962	43	0.056
	C	39.591	6.037			
**TNF-α**	M	44.616	11.265	1.652	43	0.106
	C	39.066	9.151			
**IL-17**	M	178.764	44.957	2.992	42.517	0.005^**^
	C	147.306	25.749			
**IL-10**	M	542.183	133.956	-5.47	43	0.000^***^
	C	758.875	105.015			

Student’s t-test (n = 30, X ± S), * P < 0.05, ** P < 0.01, ***P < 0.001, pg/mL; TNF-α, Tumor necrosis factor-alpha; IL, Interleukin.

## 4 Discussion

Through questionnaire investigation, we found that the oral self-evaluation results of methamphetamine users were worse than those of the healthy control group. The alterations and relations between dental plaque microbiota and oral local microenvironment of the two groups were then explored in an attempt to find a reasonable explanation. Luckily, our results demonstrated that the dental plaque microbiota of methamphetamine users showed no significant differences in alpha diversity but did exhibit differences in the relative abundance of several microbial taxa in beta diversity compared with that in the healthy controls. Furthermore, Pseudomonas and Neisseria are mainly involved in functional potential prediction of the glucose degradation (oxidative) metabolic pathway. More importantly, Neisseria, which exhibited a higher abundance in methamphetamine users, was positively correlated with the salivary level of IL-17. In contrast, Streptococcus, with a lower abundance, was positively correlated with the anti-inflammatory cytokine IL-10. To the best of our knowledge, the current study is the first to provide evidence of a link between altered oral dental plaque microbiota and systemic immuno-inflammation in methamphetamine users.

There is a relationship between differential flora and oral health status. According to our results, Proteobacteria were enriched in group M, most of which were gram-negative. This is consistent with the findings of Mok SF et al.that a significant increase in Proteobacteria was detected in patients with oral diseases compared to those in healthy individuals (20). At the genus level, *Neisseria* is a key member of the normal oral flora, which has a bidirectional effect on dental plaque. *Neisseria* synthesizes extracellular polysaccharides and glycolytic sugars in the presence of sucrose. However, the lactic acid produced by *Neisseria* can degrade plaques to form weak acids and volatile acids in the absence of sugar. *Pseudomonas* and Pasteurellaceae under Proteobacteria, are opportunistic pathogens in the oral cavity, and are closely related to the oral immune status of the host. Further, the abundance of Porphyromonas in group M was also increased significantly, and *P. gingivalis* was closely related to oral diseases. Although *P. gingivalis* is a periodontal pathogen with low abundance, alterations in its content cannot be ignored in the diseased state because its ability to cause inflammation is similar to the influence of dominant species (21). Conversely, Firmicutes were significantly decreased in group M, and *Streptococcus* and *Veillonella* were significantly decreased at the genus level. Firmicutes are the main bacterial species in the healthy oral cavity, most of which are gram-positive, with *Streptococcus* being the most abundant, followed by *Veillonella* (22). Streptococcus has been considered the dominant bacterium in the oral cavity of healthy individuals in many studies, and most of them are members of the normal flora. Concurrently, *Streptococcus* and *Veillonella* are closely synergistic; *Streptococcus* provides adhesion sites and nutrition sources for *Veillonella*, and there are signal exchanges between the two (23). *Veillonella* is a gram-negative member of the basic dental plaque microbial community. These bacteria play an important role as biological barriers in maintaining the ecological balance of the oral cavity. A rapid decrease in their content causes an imbalance in the local bacterial community. In general, gram-positive bacteria were decreased and Gram-negative bacteria were increased in the dental plaque flora of methamphetamine users. The dental plaque flora of methamphetamine users indicated a structural disorder wherein the content of symbiotic beneficial bacteria decreased sharply, and the potential pathogenic bacteria increased.

In terms of the overall trend of functional prediction, compared with the healthy controls, methamphetamine users showed greatly altered metabolic pathways with improved material metabolism and synthetic capacity in the dental plaque microecology. Carbohydrate metabolism-related functions are important in oral microecology (24). Excessive carbohydrate intake can change the local microenvironment and fermentation environment, which is conducive to the growth of cariogenic bacteria. Sustainable carbohydrate metabolism in a low-pH environment inhibits the growth of acid-sensitive bacteria and healthy bacteria (25). This contributes to the occurrence of caries, suggesting that methamphetamine abuse may have potential cariogenic effects.

The oral microbiota is closely related to the function of the human immune system, and several studies have demonstrated the relationship between oral health status and immune-related diseases, such as rheumatoid arthritis (26), dry mouth (27), systemic lupus erythematosus (28), and human immunodeficiency virus (HIV) infection (29). Most oral diseases are chronic inflammatory diseases mediated by the interaction between host immune inflammation and microorganisms. Surprisingly, these diseases intersected with the pathogenic mechanism of methamphetamine. Our results showed that the level of cytokines released in saliva had a significant correletion with the microbial community structure of dental plaque wherein the levels of inflammatory cytokine IL-1β and IL-17 in group M increased, whereas the level of the anti-inflammatory cytokine IL-10 decreased. In agreement with previously published literature, increased IL-17 is also observed in systemic lupus erythematosus(SLE) (30), rheumatoid arthritis (RA) (31)and diabetes (32) and may contribute to oral microbial alterations in these diseases. Bunte (30) described a key role for IL-17 in immune-mediated inflammatory diseases (IMIDS), such as psoriasis, rheumatoid arthritis, inflammatory bowel disease (IBD), and periodontitis. Interestingly, high levels of IL-17 have been found in patients with periodontitis, and this IL-17 induced RANKL (32). Moreover, successful treatment with anti-inflammatory drugs could partially reverse oral microbial dysbiosis. Taken together, these studies suggest that systemic disease characterized by enhanced inflammation disrupts the oral microbiota and points to IL-17 as a key mediator of this process (33). Clinically, IL-10 has also been found to be associated with immune-mediated inflammatory diseases such as inflammatory bowel disease (34), systemic lupus erythematosus (35) or rheumatoid arthritis (36), expounding the important immunomodulatory function of IL-10. Deng et al. (9)found that Lactobacillales, which show low abundance in participants with methamphetamine use disorder, were positively related to the duration of methamphetamine abstinence and the plasma level of IL-10.

In brief, systemic diseases may share common risk factors that lead to an increased inflammatory response when homeostasis is disrupted. For example, systemic diseases such as diabetes, RA, SLE and leukocyte adhesion deficiency may alter the host response to oral bacteria, resulting in a greater than normal induction of inflammation. These changes may enhance the pathogenicity of the flora and further activate inflammatory pathways. And cytokines such as IL-17 and IL-10, may play a key role in this process. That is to say, METH-induced immune changes in the body may converge to pathogenic microbial colonization, which will further affect the body’s immunity and thus cause secondary damage.

In our study, we chose supragingival plaque as the sampling point mainly considering the following reasons: Dental plaque includes supragingival plaque, adherent subgingival plaque and non-adherent subgingival plaque. Most plaque formation is supragingival plaque, which is more of a causative agent of caries, gingivitis, and supragingival tartar. And periodontal disease in a broad sense refers to a variety of pathological conditions that occur in the periodontal tissues, mainly including gum disease and periodontitis. Supragingival plaque and subgingival plaque cannot be divided intact, they are closely related. Because of the high incidence of both caries and periodontal disease in methamphetamine users, we chose supragingival plaque as an initial attempt in this experiment.

In summary, as the local oral inflammation and microorganisms are the indicators of oral health, they have certain guiding significance in the occurrence and development of methamphetamine-related oral diseases. We hope to elucidate the cause-and-effect relationship between methamphetamine, organismal immunity, and oral microbial changes in the next step of research. As a preliminary exploration, this study had some limitations, including the small number of samples and single-sex participants. All previous studies on methamphetamine and oral or fecal microbiota using 16S rRNA gene sequencing had included only male subjects, so we chose female methamphetamine users as research objects. Meanwhile, to avoid the concern of small sample size, we performed the Power calculation. The minimum number of patients required is 30 to be calculated based on 80% power and a difference of 1.09. As the difference increases, the required sample size decreases. The difference in this study was 5.95, which exceeded the minimum number of patients. Owing to the COVID-19 epidemic, oral examination was not conducted because of which direct evidence regarding oral dental plaque microbiota and oral disease could not be obtained. Concurrently, for a more rigorous study, we should single out the microflora characteristics of samples from the group with oral disease as a control to make the research theme that methamphetamine causes microflora changes by affecting inflammatory immunity more accurate. The causal relationship between alterations in oral microbiota and methamphetamine itself still remains many uncertainties. Follow-up cohort studies combined with oral examination and multiple omics analyses are thus needed to further explore the involved relationship and mechanisms. In this case, appropriate interventions and treatment measures can help improve the oral health of methamphetamine users.

## Data availability statement

The datasets presented in this study can be found in online repositories. The names of the repository/repositories and accession number(s) can be found in the article/**Supplementary Material**.

## Ethics statement

The studies involving human participants were reviewed and approved by the Ethics Committee of the School and Hospital of Stomatology, Hebei Medical University (No.2017012). The patients/participants provided their written informed consent to participate in this study. Written informed consent was obtained from the individual(s) for the publication of any potentially identifiable images or data included in this article.

## Author contributions

ZD and KG collected the samples and clinical data, performed data analysis and original draft writing. ZD and KG contributed equally to this work and share first authorship. FC revised the draft. TF and BL searched and enrolled the participants. MS and YL performed data curation. ZM conceived and designed the study. All authors have contributed to the manuscript and approved the submitted version.

## Funding

This study was supported by grants from the Beijing-Tianjin-Hebei Region’s Medical and Health Coordinated Development and Public HospitalNoReform Capacity Building of China (2019061636).

## Acknowledgments

The authors would like to thank Shanghai Personal Biotechnology Co., Ltd. (Shanghai, China) for 16S rRNA gene sequencing and providing technical support. All authors are grateful to the enrolled participants.

## Conflict of interest

The authors declare that the research was conducted in the absence of any commercial or financial relationships that could be construed as a potential conflict of interest.

## Publisher’s note

All claims expressed in this article are solely those of the authors and do not necessarily represent those of their affiliated organizations, or those of the publisher, the editors and the reviewers. Any product that may be evaluated in this article, or claim that may be made by its manufacturer, is not guaranteed or endorsed by the publisher.
