# Supplementary material for: Altered dental plaque microbiota correlated with salivary inflammation in female methamphetamine users
Source: Front Immunol. 2022 Nov 28;13:999879. doi: 10.3389/fimmu.2022.999879 (PMC9745308; doi:10.3389/fimmu.2022.999879)
Supplement: Supplementary file 1 [file DataSheet_1.docx]

Supplementary Material

# Supplementary Figures and Tables

# **Supplementary Table 1.** Demographic and clinical characteristics of study participants.

|  | **Group M** | **Group C** | **Pvalue** |
| --- | --- | --- | --- |
| **Subjects(n**) | 30 | 15 | - |
| **Age.years.Median(IQR)** | 36.50(17.25) | 33.00(21.00) | 0.335^a^ |
| **Education.years.Median(IQR)** | 9.00(0) | 12(7.00) | 0.010^a^ |
| **Duration of METH use(years;means±SD) (range)** | 6.24±3.90(2-25) | NA | - |
| **Smoke.No.(%)** | 25(83.33%) | 1(6.67%) | 0.000^b^ |
| **Brush teeth≥Twice a day.No.(%)** | 9(30.00%) | 7(46.67%) | 0.271^b^ |
| **Brushing time≥2min.No.(%)** | 12(40.00%) | 8(53.33%） | 0.396^b^ |
| **Oral self-evaluation(mcq).No.(%)** |  |  |  |
| Loose teeth, (food debris) get stuck between the teeth | 17（56.67%） | 5（33.33%） | 0.140^b^ |
| Bleeding, swollen and sore gums | 13（43.33%） | 6（40.00%） | 0.831^b^ |
| Toothache | 8（26.67%） | 2（13.33%） | 0.526^c^ |
| Oral ulcers, bad breath, dry mouth | 19（63.33%） | 3（20.00%） | 0.006^b^ |

Data are presented as Median (IQR, inter-quartile range), or N (%). ap values based on Mann-Whitney U test; bp values based on Pearson chi-square test; cp values based on Continuity Correction test; NA, not applicable.


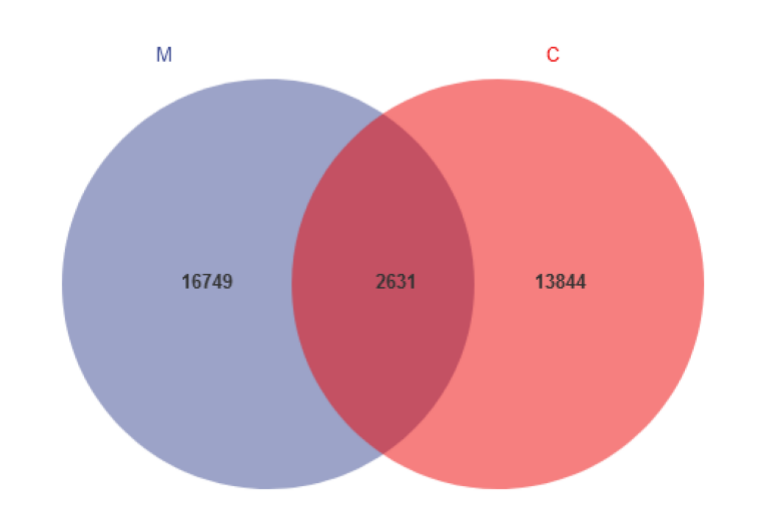


**Supplementary Figure 1.** Venn diagram showing common and unique sets of ASVs between the two groups. M, methamphetamine user group; C, control group.

**Supplementary Table 2.** Differences between groups were analyzed by PERMANOVA (***P < 0.001).

| Distance Algorithm | Group 1 | Group 2 | Sample size | Permutations | PseudoF | P value | Q value |
| --- | --- | --- | --- | --- | --- | --- | --- |
| Unweighted_unifrac | M | C | 45 | 999 | 4.070 | 0.001^***^ | 0.001 |
| Weighted_unifrac | M | C | 45 | 999 | 9.622 | 0.001^***^ | 0.001 |
